# Primary health care contributions to universal health coverage, Ethiopia

**DOI:** 10.2471/BLT.19.248328

**Published:** 2020-09-28

**Authors:** Yibeltal Assefa, Peter S Hill, Charles F Gilks, Mengesha Admassu, Dessalegn Tesfaye, Wim Van Damme

**Affiliations:** a School of Public Health, The University of Queensland, 266 Herston Road, Herston, QLD 4006, Brisbane, Australia.; b International Institute of Primary Health Care, Addis Ababa, Ethiopia.; c United States Agency for International Development, Addis Ababa, Ethiopia.; d Department of Public Health, Institute of Tropical Medicine, Antwerp, Belgium.

## Abstract

Many global health institutions, including the World Health Organization, consider primary health care as the path towards achieving universal health coverage (UHC). However, there remain concerns about the feasibility and effectiveness of this approach in low-resource countries. Ethiopia has been implementing the primary health-care approach since the mid-1970s, with primary health care at the core of the health system since 1993. Nevertheless, comprehensive and systemic evidence on the practice and role of primary health care towards UHC is lacking in Ethiopia. We made a document review of publicly available qualitative and quantitative data. Using the framework of the Primary Health Care Performance Initiative we describe and analyse the practice of primary health care and identify successes and challenges. Implementation of the primary health-care approach in Ethiopia has been possible through policies, strategies and programmes that are aligned with country priorities. There has been a diagonal approach to disease control programmes along with health-systems strengthening, community empowerment and multisectoral action. These strategies have enabled the country to increase health services coverage and improve the population’s health status. However, key challenges remain to be addressed, including inadequate coverage of services, inequity of access, slow health-systems transition to provide services for noncommunicable diseases, inadequate quality of care, and high out-of-pocket expenditure. To resolve gaps in the health system and beyond, the country needs to improve its domestic financing for health and target disadvantaged locations and populations through a precision public health approach. These challenges need to be addressed through the whole sustainable development agenda.

## Résumé

De nombreux organismes sanitaires internationaux, dont l'Organisation mondiale de la Santé, considèrent que les soins de santé primaires représentent un jalon essentiel du cheminement vers une couverture maladie universelle (CMU). Néanmoins, des inquiétudes subsistent quant à la faisabilité et à l'efficacité de cette approche dans les pays à faible revenu. L'Éthiopie a adopté cette approche depuis le milieu des années 1970, et les soins de santé primaires figurent au cœur du système sanitaire depuis 1993. Pourtant, rares sont les preuves exhaustives et généralisées concernant la pratique et le rôle des soins de santé primaires dans l'évolution vers une CMU en Éthiopie. Nous avons donc procédé à un examen des documents contenant des données qualitatives et quantitatives accessibles au public. À l'aide du cadre instauré par l'initiative PHCPI (Primary Health Care Performance Initiative), nous avons décrit et analysé la pratique des soins de santé primaires; nous avons également identifié les réussites et les défis. C'est grâce à des politiques, stratégies et programmes en adéquation avec les priorités nationales que cette méthode axée sur les soins de santé primaires a pu être mise en œuvre en Éthiopie. Le pays a emprunté une approche diagonale vis-à-vis des programmes de contrôle des maladies, tout en renforçant les systèmes de santé, l'autonomie des communautés et l'action multisectorielle. Ces stratégies lui ont permis d'augmenter la prise en charge des services de santé et d'améliorer l'état de santé de la population. Il reste cependant d'importants défis à relever: couverture insuffisante des services, inégalités d'accès, lenteur de transition entre systèmes sanitaires pour la fourniture de prestations liées aux maladies non transmissibles, qualité médiocre des soins et frais non remboursables élevés. Afin de combler les lacunes au sein et en dehors du système de santé, le pays doit revoir le montant du financement octroyé aux soins de santé à la hausse, mais aussi cibler les régions et populations défavorisées par le biais d'une approche de précision en matière de santé publique. Ces défis doivent être abordés tout au long du programme de développement durable.

## Resumen

Varias instituciones sanitarias mundiales, incluida la Organización Mundial de la Salud, opinan que la atención primaria de salud es la vía hacia el logro de la cobertura sanitaria universal (CSU). Sin embargo, persisten las preocupaciones acerca de la viabilidad y la eficacia de este enfoque en los países de bajos recursos. Etiopía aplica el enfoque de la atención primaria de salud desde mediados del decenio de 1970, por lo que la atención primaria de salud es el núcleo del sistema de salud desde 1993. No obstante, Etiopía carece de pruebas integrales y generales sobre la práctica y la función de la atención primaria de salud orientada a la CSU. Se realizó un análisis documental de los datos cualitativos y cuantitativos a disposición del público. Se describe y analiza la práctica de la atención primaria de salud y se determinan los éxitos y los desafíos por medio del marco de la Iniciativa sobre el desempeño de la atención primaria de salud. La aplicación del enfoque de la atención primaria de salud en Etiopía fue posible gracias a las políticas, las estrategias y los programas que se adaptaron a las prioridades del país. Existe un enfoque diagonal de los programas de control de enfermedades sumado al fortalecimiento de los sistemas sanitarios, a la participación de la comunidad y a las medidas multisectoriales. Gracias a estas estrategias, el país ha logrado aumentar la cobertura de los servicios sanitarios y mejorar el estado de salud de la población. Sin embargo, aún quedan por resolver algunos desafíos fundamentales, como la cobertura insuficiente de los servicios, la falta de equidad en el acceso, la lentitud de la transición de los sistemas sanitarios para prestar los servicios correspondientes a las enfermedades no transmisibles, la calidad deficiente de la atención y los elevados gastos de bolsillo. Para resolver las deficiencias del sistema sanitario y otros aspectos, el país debe mejorar su financiamiento nacional para la salud y centrarse en los lugares y las poblaciones desfavorecidos a través de un enfoque preciso de la salud pública. Se debe abordar estos desafíos en todo el programa de desarrollo sostenible.

## ملخص

إن العديد من المؤسسات الصحية العالمية، بما في ذلك منظمة الصحة العالمية، تعتبر الرعاية الصحية الأولية بمثابة الطريق نحو تحقيق التغطية الصحية الشاملة (UHC). وعلى الرغم مع ذلك، لا تزال هناك مخاوف بشأن جدوى وفعالية هذا الأسلوب في البلدان منخفضة الموارد. استمرت إثيوبيا في تنفيذ أسلوب الرعاية الصحية الأولية منذ منتصف السبعينيات، مع وضع الرعاية الصحية الأولية في صميم النظام الصحي منذ عام 1993. وعلى الرغم من ذلك، يفتقر الأمر لوجود أدلة شاملة ومنهجية على ممارسة الرعاية الصحية الأولية ودورها تجاه التغطية الصحية الشاملة (UHC). لقد قمنا بإجراء مراجعة لوثائق البيانات النوعية والكمية المتاحة للجمهور. باستخدام إطار عمل مبادرة أداء الرعاية الصحية الأولية، نقوم بوصف ممارسة الرعاية الصحية الأولية وتحليلها، مع تحديد النجاحات والتحديات. أصبح تنفيذ أسلوب الرعاية الصحية الأولية في إثيوبيا ممكناً من خلال السياسات والاستراتيجيات والبرامج التي تتماشى مع أولويات الدولة. كان هناك أسلوب قُطري لبرامج مكافحة الأمراض، إلى جانب تعزيز النظم الصحية، وتمكين المجتمع والعمل متعدد القطاعات. أدت هذه الاستراتيجيات إلى تمكين الدولة من زيادة تغطية الخدمات الصحية، وتحسين الوضع الصحي للسكان. ومع ذلك، فلا تزال هناك تحديات رئيسية يجب التعامل معها، بما في ذلك التغطية غير الكافية للخدمات، وعدم المساواة في الحصول على هذه الخدمات، والتحول البطيء للنظم الصحية لتقدم خدمات للأمراض غير المعدية، وسوء جودة الرعاية، وارتفاع الإنفاق الشخصي. ولسد الثغرات في النظام الصحي وما عداه، تحتاج الدولة إلى تحسين تمويلها المحلي للصحة، واستهداف المواقع المضارة والسكان المحرومين من خلال أسلوب دقيق للصحة العامة. تحتاج هذه التحديات للمواجهة من خلال جدول أعمال كامل للتنمية المستدامة.

## 摘要

包括世界卫生组织在内的众多全球卫生机构都认为，初级卫生保健是实现全民健康覆盖 (UHC) 的途径。然而，在资源匮乏国家这种方式的可行性和有效性仍备受关注。埃塞俄比亚自上世纪 70 年代中期以来一直推行初级卫生保健，且从 1993 年开始，初级卫生保健就成为其卫生系统的核心。但埃塞俄比亚缺乏其推行初级卫生保健及初级卫生保健对实现 UHC 作用的全面和系统依据。我们对公开发布的定性和定量数据进行了文件审查。使用初级卫生保健实施方案的框架，我们描述并分析初级卫生保健的推行方法，从而确定其成功经验及面临的挑战。通过与国家优先事项一致的政策、战略和计划，埃塞尔比亚实施推行初级卫生保健。在强化卫生系统、社区授权和跨部门合作的同时，还可以对疾病防控计划采取对角线方法。国家可通过这些战略增加卫生服务覆盖面并改善国民健康状况。但仍面临着一些关键挑战，如服务覆盖面不足、获取服务机会不平等、卫生系统转型速度慢不足以提供非传染性疾病医疗服务、医疗质量不到位以及自费部分费用高昂。为解决卫生系统及其他方面的不足之处，国家需要改善国内卫生融资，并通过精确的公共卫生方式精准定位弱势地区和群体。这些挑战需凭借可持续发展整体议程逐一击破。****

## Резюме

Множество глобальных учреждений здравоохранения, включая Всемирную организацию здравоохранения, рассматривают первичную медико-санитарную помощь как основную составляющую обеспечения всеобщего охвата услугами здравоохранения (ВОУЗ). Однако сохраняются опасения относительно осуществимости и эффективности такого подхода в странах с низким уровнем ресурсов. Эфиопия применяет подход внедрения первичной медико-санитарной помощи с середины 1970-х годов, при этом первичная медико-санитарная помощь занимает центральное место в системе здравоохранения с 1993 года. Тем не менее в Эфиопии отсутствуют исчерпывающие и системные данные о практике и роли первичной медико-санитарной помощи в обеспечении всеобщего охвата услугами здравоохранения. Авторы подготовили документальный обзор общедоступных качественных и количественных данных. Используя структуру Инициативы по эффективной первичной медико-санитарной помощи, авторы описывают и анализируют практику первичной медико-санитарной помощи, а также определяют успехи и проблемы этого процесса. Внедрение подхода первичной медико-санитарной помощи в Эфиопии стало возможным благодаря политике, стратегиям и программам, согласующимся с приоритетами страны. К программам борьбы с болезнями применялся диагональный подход наряду с укреплением систем здравоохранения, расширением прав и возможностей сообществ и многосекторальными действиями. Эти стратегии позволили стране расширить охват услугами здравоохранения и улучшить состояние здоровья населения. Тем не менее ключевые проблемы еще предстоит решить, включая недостаточный охват услугами, неравенство в доступе к медицинской помощи, медленный переход систем здравоохранения к оказанию услуг по лечению неинфекционных заболеваний, ненадлежащее качество медицинской помощи и высокий уровень расходов из собственных средств населения. Для устранения пробелов в системе здравоохранения и за ее пределами стране необходимо улучшить внутреннее финансирование здравоохранения и сосредоточить внимание на неблагополучных районах и группах населения посредством прицельного подхода к общественному здравоохранению. Эти проблемы необходимо решать в рамках повестки дня в области обеспечения устойчивого развития в целом.

## Introduction

Universal health coverage (UHC) is the main target of sustainable development goal (SDG) 3, i.e. to ensure healthy lives and promote well-being for all at all ages. UHC is about giving all people access to quality health services according to need, while also ensuring that the use of these services does not expose the user to financial hardship.^1^^,^^2^ Although there is consensus about why and what is needed for UHC, there is debate about how to achieve it.^3^ Over the past 40 years, primary health care has been shown to increase access to services, improve service coverage and quality in the most efficient and equitable way, and contribute to financial protection for individuals and households.^4^^–^^7^ Many of those involved in global health, including the World Health Organization, consider that primary health care is the path towards achieving UHC.^8^^–^^10^

Following the Astana Declaration in 2018,^11^^,^^12^ there is increased interest from countries to transform their primary health-care systems towards UHC.^13^ However, the aspiration to UHC will be realized only if there is a political commitment that will be translated into three interrelated and synergistic pillars of primary health care: (i) universal access to quality health services (supported by essential public health functions) and equity of access to health care; (ii) empowered people and communities; and (iii) multisectoral policy and action for health.^6^^,^^14^ There are still concerns, however, about the feasibility and effectiveness of the primary health-care approach towards UHC in low-resource countries. Moreover, there is inadequate evidence on the policy and practice of primary health care and UHC in these countries.^12^^,^^15^

Ethiopia has been implementing the primary health-care approach since the mid-1970s when it developed its health policy that emphasized disease prevention and control, gave priority to rural areas and advocated for community involvement.^16^ Since 1993 primary health care has been the core of the country’s health system.^16^ The structure and composition of the system has evolved over time and currently consists of primary hospitals, health centres and health posts. Primary hospitals provide promotive, preventive, curative and rehabilitative outpatient care, basic emergency surgical procedures, and comprehensive emergency obstetric care, with a minimum capacity of 35 beds. Health centres provide promotive, preventive, curative and rehabilitative outpatient care, and inpatient care with the capacity of 10 beds for emergency and delivery services. Health posts provide essential promotive and preventive services and limited curative services.^17^

Despite consistent implementation of this approach in Ethiopia over the past three decades, comprehensive and systemic evidence on the practice and role of primary health care towards UHC is lacking in the country.^17^ We carried out a policy evaluation to identify the successes and challenges towards achieving UHC in Ethiopia.

## Conceptual framework

We made a review of publicly available documents to describe and analyse the practice of Ethiopia’s primary health-care approach (Box 1; available at: http://www.who.int/bulletin/volumes/98/12/19-248328). We used the five domains of the Primary Health Care Performance Initiative conceptual framework to guide the extraction and analysis of quantitative and qualitative data: (i) policy and system; (ii) inputs; (iii) contextual factors (community empowerment and multisectoral action); (iv) service delivery; and (v) outcomes.^19^^,^^20^ An advantage of the framework is that it is consistent with the three pillars of primary health-care systems: political commitment; community empowerment; and universal access and equity. The framework is based on existing frameworks for health systems, describes all the important components of a strong primary health-care system, and provides indicators to inform and drive efforts to improve primary health care.^19^^,^^20^ We also analysed data on multisectoral action to improve the social determinants of health.^6^^,^^14^

Box 1 **Data sources and methods of analysis for the policy review of primary health care, Ethiopia**

We conducted document reviews using a parallel mixed-methods design.^18^ We used publicly available secondary data sources, including government offices (the Ethiopian Federal Ministry of Health, Federal HIV/AIDS Prevention and Control Office, Ethiopian Public Health Institute and Ethiopian Central Statistics Authority) and WHO. We also used peer-reviewed articles, and health policy and strategy documents from the health ministry and WHO. We did not include documents published before 1990.^16^

We collected data using the framework and tools developed by the Primary Health Care Performance Initiative.^19^^,^^20^ We extracted quantitative and qualitative data on the successes and challenges in the practice of primary health care using data extraction tools on policy and strategy, community ownership and multisectoral action. We extracted quantitative data on the successes and challenges in the inputs, the three dimensions of UHC – populations covered, services covered and costs covered over time – and health outcomes.

We used a qualitative and interpretive thematic synthesis approach to identify and synthesize policies, strategies and programmes in the practice of primary health care in Ethiopia over time, and to summarize successes and challenges towards UHC.^21^ A trend analysis of the quantitative data was carried out to check for changes in health services coverage and health outcomes over time. We undertook equity analysis to check for changes in health services and health outcomes. We measured coverage as percentage of population using services. We used rate ratios (calculated as rate in a given region divided by rate in the capital city, Addis Ababa) to measure inequality in health service coverage and health status.^22^

AIDS: acquired immunodeficiency syndrome; HIV: human immunodeficiency virus; UHC: universal health coverage; WHO: World Health Organization.

## Policy and system 

### Country leadership and governance

Ethiopia’s current health policy was inaugurated in 1993 with the aim of increasing access to primary health-care services.^16^ The policy has five pillars: (i) democratization and decentralization of the health system; (ii) preventive and promotive health services; (iii) access to health care for all the population; (iv) intersectoral collaboration; and (v) enhancing national self-reliance by mobilizing and efficiently utilizing resources for health. A sixth element of the policy is to consider broader issues such as population, food, living conditions and other essentials of life for better health.^16^

Box 2 summarizes the major health policies, strategies and their components towards universal access to health services since 1990. Ethiopia’s first 20-year health sector development programme was developed and implemented in four phases from 1995–2015 to translate the policy into actions.^23^^–^^26^ In 2015, the Ethiopian government introduced its second 20-year strategy towards UHC through strengthening of primary health care.^28^ The health sector transformation plan, which aligns well with SDG 3, aims to build the health system capacity and improve UHC.^27^ The current phase aims to expand coverage of services for noncommunicable diseases and mental health. The transformation plan has identified four interrelated agendas: (i) quality and equity of health care; (ii) district transformation; (iii) compassionate, respectful and caring health professionals; and (iv) information revolution.^27^

Box 2 **Major health policies, strategies and their components towards universal access to health services, Ethiopia, 1990–2020**

***Since 1990***

**Health policies^16^**

Democratization and decentralization of the health system; preventive and promotive health services; access to health care by whole population; intersectoral collaboration; enhancing national self-reliance by mobilizing and efficiently using resources for health; and consider broader issues, e.g. population, food, living conditions and other essential needs for better health.

***1995–2005***

**Health sector development plan phases 1 and 2^23^^–^^25^**

Improve health service delivery; enhance health facility expansion; develop human resources; strengthen pharmaceutical supply and management; improve information, education and communication; enhance health sector management; strengthen health management information systems; improve health-care financing; and create health extension programme.

***2006–2010***

**Health sector development plan phase 3^23^^–^^25^**

Improve health service delivery and quality of care; enhance integrated disease surveillance and public health emergency management; strengthen health extension programme; reform the health sector: business process re-engineering; reinforce health facility construction and expansion; develop human resource; enhance pharmaceutical services; improve health and health related services and products regulation; strengthen governance, including harmonization and alignment; implement one-plan, one-budget, one-report strategy; improve health care financing; enhance retention and utilization of revenue; improve health insurance; and strengthen operational research.

***2011–2015***

**Health sector development plan phase 4^26^**

Improve access to and quality of health services; improve community ownership; maximize resource mobilization and utilization; improve public health emergency preparedness and responses; improve pharmaceutical supply and services; improve regulatory system; improve evidence-based decision-making, including harmonization and alignment; improve health infrastructure; and improve human capital and leadership.

***Since 2016***

**Health sector transformation plan^27^**

Enhance community ownership, participation and engagement; improve equitable access to quality health services; improve health emergency risk management; enhance good governance; improve regulatory system; improve supply-chain and logistics management; improve resource mobilization; improve research and evidence for decision-making; advance use of technology and innovation; improve development and management of human resource; develop health infrastructure; enhance policy and procedures; strengthen transformation agendas; and establish the International Institute for Primary Health Care.

The health sector has also implemented several reforms, such as the business process re-engineering, aiming to improve community satisfaction, scale-up health services and enhance the quality of care. The reforms have increasingly decentralized management of the primary health-care system and created opportunities for governance at local levels to improve the effectiveness, efficiency, equity and sustainability of health services. 

Bilateral and multilateral development partners have made an important contribution towards universal access to health services. These successes have been facilitated by harmonization and alignment of the different activities in a one-plan, one-budget, one-report approach.^29^ Ethiopia was one of the signatories of the International Health Partnership + Global Compact and the first country to develop and sign a country-based compact. Ethiopia’s Joint Consultative Forum has promoted harmonization and alignment of programme activities, mobilization of resources, and implementation and monitoring.^27^ A joint financing arrangement was also established to manage the millennium development goals (MDGs) performance fund, which is a pooled funding mechanism for programme areas in the primary health-care system. The fund is supported by development partners, including the World Bank and European Union.^30^

### Community health programme

Ethiopia’s health extension programme was launched in 2003 with the mission to deliver 16 packages of health promotion, disease prevention and basic curative services closer to the community (Box 3). The programme has enabled Ethiopia to achieve significant improvements in maternal and child health; prevention and control of communicable diseases; hygiene and sanitation; knowledge and health-care seeking; and community engagement.^17^

Box 3 **Example of community health programme in Ethiopia**

Ethiopia’s health extension programme was launched in 2003 with the mission to deliver 16 packages of health promotion, disease prevention and basic curative services closer to the community. The programme focuses on four areas: (i) promotion of hygiene and environment sanitation; (ii) prevention and control of major communicable diseases; (iii) promoting and providing family health services; and (iv) health education and communication.^17^

Over 38 000 female health extension workers from their communities were trained in regional institutions for one year. Two health extension workers were deployed to each health post to serve 5000 people. Health extension workers provide services (family planning, maternity services, immunizations and nutrition counselling) in the health post (25% of their work time) and in the community (75% of their work time), referring patients with more complex health needs to health centres and collecting vital statistics in the community.^23^ Assessment of community perspectives on primary health care indicated that there was a positive attitude towards the service offered by health extension workers,^31^ reported high satisfaction (mean score 83.0 out of 100; standard deviation: 18.2) and favourable interpersonal relationships (75.5% of the 379 study participants).^32^^,^^33^

Despite these successes, the programme has faced challenges, including resource gaps (medical equipment and drugs); limited supportive supervision; absence of a well-established referral system; high turnover of health extension workers; absence of a clear career structure for health extension workers and unattractive salary scale. There were also community complaints about inadequate curative care and delivery services.^34^ These challenges remain to be addressed for progress in UHC.^17^

## Inputs 

**Fig. 1:**
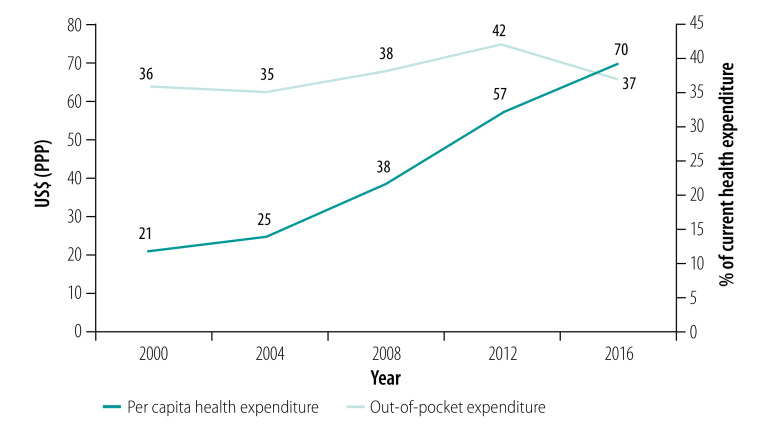
Per capita health expenditure and out-of-pocket health expenditure in Ethiopia, 2000–2016

Ethiopia has demonstrated a strong commitment to health-systems strengthening. The health sector development programme introduced reforms to increase financing for health. The proportion of health financing from domestic sources (excluding contribution from donors) has increased from 53% of United States dollars (US$) 1.3 billion in 2008 to 78% of US$ 2.7 billion in 2017.^35^ The per capita spending on health increased from US$ purchasing power parity (PPP) 21 in 2000 to US$ PPP 70 in 2016 (Fig. 1). Most of the expenditure occurs at primary health-care level.^36^^,^^37^ In 2013–2014, primary health care received 54% (US$ 59 million) of the total recurrent government expenditure of US$ 110 million, 43% (US$ 47 million) of which was spent on health centres and health posts.^35^

US$ (PPP): United States dollars (purchasing power parity).

Source: Ethiopian Federal Ministry of Health; 2017.^35^

The total number of health centres and hospitals increased from 775 in 2005 to 1463 in 2012 and 3858 in 2015. To address its human resources for health gaps, the number of health professional training schools has increased exponentially since 2008.^36^ There has also been parallel expansion in enrolment and graduation outputs. The human resources for health density increased from 8.4 to 13 per 10 000 population between 2008 and 2013.^27^ The population per doctor dropped from 37 000 in 2000 to 24 000 in 2019.^38^^,^^39^

Ethiopia established the Pharmaceutical Fund and Supply Agency in 2007 to enhance procurement and distribution of drugs.^40^ The agency has increased its annual distribution capacity sixfold between 2010 and 2015, with national storage capacity raised from 46 260 m^3^ to 531 000 m^3^ and the cold-chain storage capacity from 50 m^3^ to 800 m^3^. A national survey conducted in 2014 indicated that the average availability of tracer essential medicines at health facilities increased from 65% of essential medicines before the establishment of the agency to 89% of essential medicines in 2014.^36^

Ethiopia has improved its health management information system since 2008, providing information for planning, monitoring and resource use. As an extension of the system, the rollout of family folders is of particular interest for strong primary health care systems. The system provides opportunities to strengthen evidence-based planning, service delivery, monitoring and evaluation. Family folders are also used to establish the vital statistics registration system in the country.^41^

Ethiopia implemented a mix of vertical and horizontal approaches to strengthen its health systems and to scale-up disease control programmes concurrently, mobilizing resources from targeted programmes and investing them in health-systems strengthening. Global health initiatives, such as the Global Fund to Fight AIDS, Tuberculosis and Malaria, the President’s Emergency Plan for AIDS Relief, and the Global Alliance for Vaccines Initiative, have made important contributions to health-systems strengthening and scaling-up priority services.^36^

Despite these successes in health systems inputs, several challenges remain. According to an assessment in 2016, only 54% of 547 health facilities, excluding health posts, were ready to provide general health services.^42^ There was suboptimal service availability and readiness, limited integrated service delivery, and inadequate referral and feedback systems. There was also maldistribution of skilled human resources, a high attrition rate of health workers and inadequate motivation among staff. Documentation, dissemination and use of evidence from the routine health management information system and research data were suboptimal. There were supply-chain gaps in forecasting, distribution and availability of medical equipment, inadequate maintenance of equipment and low utilization of technology and innovations in appropriate technology.^26^^,^^36^

The primary health-care system has also faced gaps in financing due to decreased overseas development assistance, in addition to inadequate capacity for resource mobilization and utilization. Out-of-pocket expenditure is persistently high and reached close to 40% of current health expenditure of US$ 2.6 billion in 2016 (Fig. 1). The incidence of catastrophic health expenditure, at the threshold of 10% of household total consumption, was 4.9% in 2015.^43^ In April 2001, Heads of State of African Union countries pledged to set a target of allocating at least 15% of their annual budget to the health sector. However, Ethiopia is far from achieving this target, as only 6% of the government’s general expenditure of US$ 8 billion in 2016 was allocated to health.^35^ The country has been mindful of this gap and has introduced several health-care financing initiatives, although the scale and speed of implementation is lagging.^26^^,^^36^^,^^44^

We also identified inadequate implementation of policies and programmes at all levels of the health system. These challenges vary across regions in the country.^26^^,^^36^ Gini indices consistently revealed high overall inequalities in health expenditure, health workforce and infrastructure among regions. These variations can explain the regional differences in health services delivery and health outcomes.^44^

## Contextual factors

### Community empowerment

Community ownership has been central in the design, implementation and monitoring and evaluation of strategies and programmes. Ethiopia’s government has used two strategies to enhance community participation and ownership: the creation of model families; and the health development army (Box 4).^45^^–^^48^ These strategies aim to engage communities, identify locally prominent challenges that hinder uptake of services, and scale-up best practices.^27^

Box 4 **Example of two strategies to enhance community participation and ownership in Ethiopia**

Model families and the health development army facilitate innovation, diffusion and behaviour change communication, and help improve service delivery. One example is increasing demand for HIV testing and reduction of new HIV infections facilitated by community conversations. Model families and the health development army have also helped to increase delivery in a health facility from below 20% of 0.64 million births in 2011 to nearly 60% of 2.04 million births in 2015 by creating demand, introducing mother-friendly practices and contributing resources such as the construction of maternity waiting homes.^45^ The development army is an important source of information for mothers to prepare themselves for birth and related complications. Well established groups have strengthened the linkage of the health facility to the community and minimized delays in maternal health service use.^46^ In addition, the community has participated in the expansion of primary health care by leveraging domestic resources through providing matching funds and labour for the construction of health centres and health posts.^47^^,^^48^

HIV: human immunodeficiency virus.

Despite these successes, several challenges have hampered the implementation of initiatives to enhance community participation and ownership towards UHC and improved health status. The implementation of initiatives to increase community mobilization are directly and indirectly affected by social, cultural, political and economic determinants that underpin health. There is a perception by some communities that these initiatives, mainly the health development army, have more of a political role than a health promotion and disease prevention role. The implementation of these initiatives in pastoralist regions (Afar, Benishangul-Gumuz, Gambela and Somali) is still at an early stage, due to inadequate capacity and political commitment at regional and district levels. This delay is reflected in inequities in access and utilization of health services and health outcomes across Ethiopia.^30^^,^^46^

### Multisectoral action

Health depends not only on access to health care but also on financial resources, education and access to basic utilities such as water and roads.^49^ Ethiopia has designed and implemented policies and strategies to guide its economic development, including poverty reduction strategies framed in the context of the MDGs and the SDGs. Five-year development plans including multiple sectors are aimed at improving economic growth.^50^ The plans have promoted increased capacity of the health system through improvements in infrastructure and training of health workers.^50^

**Fig. 2:**
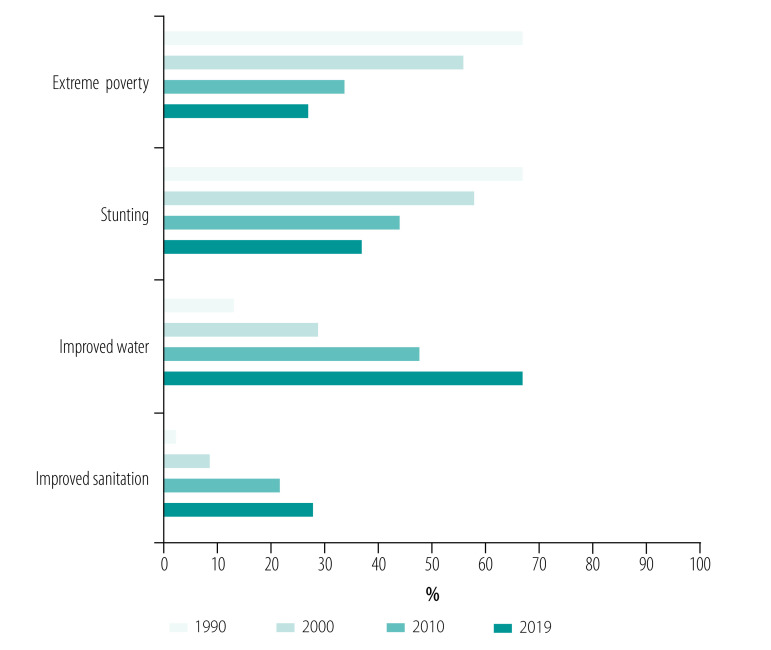
Trends in the level of social determinants of health in Ethiopia, 1990–2019

Notes: Extreme poverty: % of people living on 1.25 United States dollars or less a day; stunting: % of children under age 5 years who are stunted; improved water: % of households with improved water; improved sanitation: % of households with improved sanitation.

Sources: Haile T; 2015.^51^ Central Statistical Agency and ICF International; 2016.^52^ Ethiopian Public Health Institute and ICF International; 2019.^53^

Ethiopia has achieved strong economic growth and expanded social services over the past three decades. Per capita income has tripled from US$ 254 in 1990 to US$ 772 in 2019. The level of extreme poverty (proportion of people living on US$ 1.25 or less a day) declined from 67% of the population of 48 million in 1990 to 27% of 112 million in 2019 (Fig. 2).^51^ Access to social services increased: percentage of eligible children in primary education reached 100%; secondary school enrolment increased from 12% in 1990 to 38% in 2019%; the proportion of women with no education decreased from 66% in 2005 to 48% in 2016; use of improved water increased from 13% of households in 1990 to 67% in 2019 (Fig. 2).^52^ The proportion of households practising open defecation dropped from 82% in 2000 to 32% in 2016.^54^ The total fertility rate declined from 5.5 to 4.6 children per woman between 2000 and 2016.^52^

Despite these improvements in the social determinants of health, the same data reveal gaps remaining towards UHC (Fig. 2). In 2019, more than a quarter of the population were living under extreme poverty. More than one-third of the 16 million children (37%) were affected by chronic malnutrition (stunted). Access to improved water was inadequate (67% of households) and improved sanitation was very low (27% of households). There were also constraints in coverage and quality of roads.^55^ Lack of peace and stability in the country over the last five years has hampered the implementation of the primary health-care approach.^56^ Moreover, the multisectoral activities that are essential to strengthen the implementation of primary health care towards UHC are inadequately coordinated.^38^

The social determinants of health vary across regions (Fig. 2).^52^^,^^57^ In 2016 total fertility rate was highest in Somali (7.2 children per woman) and lowest in Addis Ababa (1.8 children per woman).^52^ In the same year, less than 10% of rural households relied on unimproved sanitation while nearly half of urban households had access to improved sanitation.^52^ These variations in social determinants of health can explain the inequity in health services delivery and health outcomes among regions in the country.

## Service delivery

Ethiopia has implemented a set of effective maternal and child health interventions, including family planning, antenatal care, skilled birth attendance, postnatal care and immunization. The government has also emphasized the prevention and control of infectious diseases, including human immunodeficiency virus (HIV), tuberculosis and malaria.

**Fig. 3:**
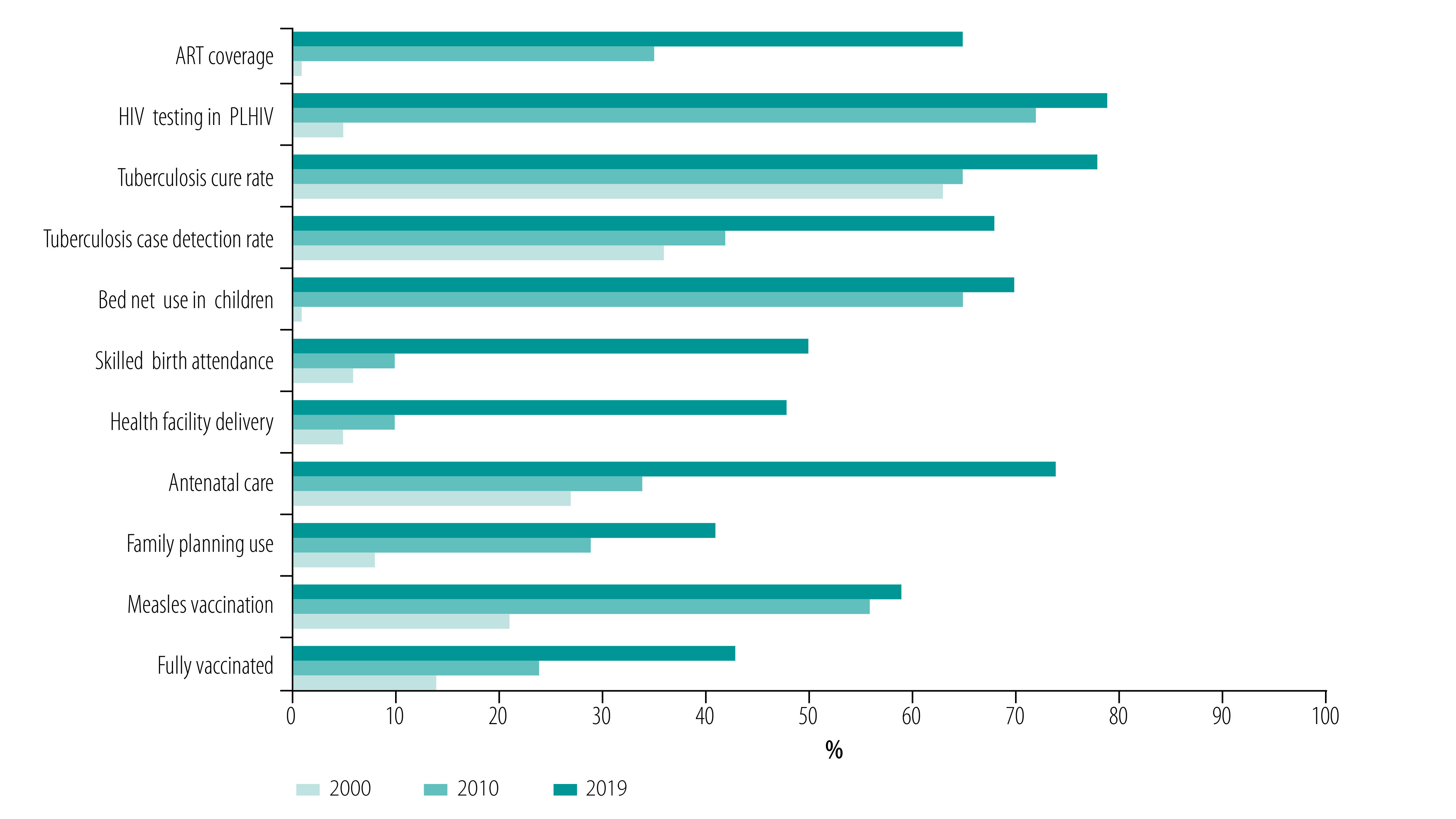
Trends in coverage of priority health services in Ethiopia, 2000–2019

ART: antiretroviral therapy; HIV: human immunodeficiency virus; PLHIV: people living with HIV.

Notes: Fully vaccinated: % of living children aged 12–23 months who are fully vaccinated; measles vaccination: % of living children aged 12–23 months who are vaccinated for measles; family planning use: % of currently married women aged 15–49 years who use family planning methods; antenatal care: % of pregnant women who received antenatal care from skilled providers, such as doctors and nurses or midwives, health officers, and health extension workers; health facility delivery: % of deliveries that occur in a health facility; skilled birth attendance: % of births delivered with the assistance of doctors, nurse or midwives, health officers, and health extension workers; bed net use in children: % of children younger than 5 years who use bed net; tuberculosis case detection rate: % of new and relapse tuberculosis cases notified out of estimated cases during the year; tuberculosis cure rate: % of cure in notified new and relapse tuberculosis cases; HIV testing in PLHIV: % of people living with HIV who know their HIV status; ART coverage: % of people living with HIV who are taking ART.

Sources: Ethiopian Federal Ministry of Health; 2016.^27^ Central Statistical Agency and ICF International, 2016.^52^ Ethiopian Public Health Institute and ICF International, 2019.^53^

A service provision assessment in 2014 found that more than 90% of the primary hospitals and health centres provided the full package of services related to maternal and child health and HIV, tuberculosis and malaria prevention and control. More than 80% of health posts provided child health, family planning and antenatal care services. As a result, health services coverage of priority programmes increased significantly between 2000 and 2019 (Fig. 3).^27^ Measles vaccination in children aged 12–23 months increased from 21% in 2000 to 59% in 2019. The proportion of births assisted by a skilled birth attendant increased from 6% in 2000 to 50% in 2019.^52^ The proportion of children sleeping under a bed net increased from 1% in 2000 to 70% in 2019. Tuberculosis case detection increased from 36% in 2000 to 68% in 2019. The proportion of people living with HIV on antiretroviral therapy increased from 1% in 2000 to 65% in 2019.^27^^,^^54^^,^^58^

Despite the overall progress in health services delivery, Ethiopia still falls short of UHC. In 2019 the UHC service coverage index was still very low at 39%.^43^^,^^57^ The index is based on tracer interventions for reproductive, maternal, newborn and child health, infectious diseases and noncommunicable diseases. Fig. 3 and Table 1 show that there are still big gaps in coverage of priority programmes.^54^ In addition, the overall UHC service coverage indices vary across regions, from 10% in Afar to 52% in Addis Ababa in 2015.^57^ In the 2016 demographic and health survey^52^ antenatal care coverage was highest in Addis Ababa (97%) and lowest in Somali (44%). Only 53% of women with no education obtained antenatal care services, compared with 98% of women with more than secondary education.^52^ Women in the highest wealth quintile (85%) were more likely than those in the lowest quintile (48%) to receive antenatal care or to be delivered by skilled providers (70% versus 11%).^52^ The proportion of women who had been tested for HIV in the previous 12 months was twice as high in urban areas (36%) as in rural areas (15%).^52^ The proportion of men tested for HIV varied from 13% among those with no education to 39% among those with more than secondary education.^52^

**Table 1:** Priority health interventions and social determinants of health in selected regions, Ethiopia, 2000 and 2016

Region	Facility delivery, % of deliveries			Measles vaccination, % of children			HIV testing, % of people			No education, % of women			Underweight, % of children	
	2000	2016		2000	2016		2000	2016		2000	2016		2000	2016
Addis Ababa	67	97		88	93		17	73		25	9		14	5
Amhara	3	27		27	62		1	53		84	54		52	28
Tigray	4	57		67	80		1	66		78	43		48	23
Somali	6	18		39	48		2	14		89	75		44	29
Oromia	4	19		20	43		2	32		76	51		42	23

HIV: human immunodeficiency virus.

Source: Central Statistical Agency and ICF International; 2016.^52^

Notes: Facility delivery: % of deliveries that occur in a health facility; measles vaccination: % of living children aged 12–23 months vaccinated for measles; HIV testing: % of women and men who were ever tested for HIV; no education: % of women with no education; underweight: % of children younger than 5 years who are underweight.

The health systems is also slowly transitioning towards management of noncommunicable diseases. Ethiopia is undergoing an epidemiological transition, with the burden of noncommunicable diseases steadily increasing due to behavioural and lifestyle changes, demographic shifts and reductions in communicable, maternal, neonatal and nutritional diseases. Noncommunicable diseases were estimated to account for 42% of total deaths of 677 045 in 2015, while the proportion of all disability adjusted life-years attributed to noncommunicable diseases increased from 20% in 1990 to 69% in 2015.^59^ Despite this fast epidemiological transition, reorienting the health system to address noncommunicable diseases is slow, with services primarily designed for the management of infectious diseases, maternal and child health problems and undernutrition.^60^ For example, Ethiopia’s UHC service coverage index in 2015 was 35% for noncommunicable diseases compared with 53% for infectious diseases.^57^ The service capacity index for noncommunicable diseases was only 20%, as resources allocated for noncommunicable diseases are very low.^57^ Service availability and readiness for noncommunicable diseases remains low.^61^ For example, less than a quarter (24%) of 547 health facilities (health centres and hospitals) in 2014 were ready to provide services for either diagnosis or treatment of diabetes, ranging from 5% in rural areas to 34% in urban areas.^42^ In 2016, there was a large gap in overall mean diagnostic capacity for noncommunicable diseases (39% of 632 health centres and hospitals) and availability of essential noncommunicable disease drugs, such as angiotensin-converting-enzyme inhibitors (25%) and insulin injection (18%).^61^ To address the gaps, the country has developed a strategic framework to improve services for the prevention and control of noncommunicable diseases.^60^

We also identified inadequate quality of care.^27^ For instance, the infrastructure expansion to improve maternal health was not translated into high quality of intrapartum care.^62^ In a 2018 study conducted in 32 primary health-care health facilities providing delivery care, the input quality was good in 66% of health facilities; however, the process and output quality was good in only 31% of health facilities.^63^ Despite these challenges, there are opportunities. Quality is included in the government’s future plans;^27^ the national health-care quality strategy has had promising results;^64^ and the use of a community-based collaborative quality improvement initiative has improved postnatal care services in rural Ethiopia.^65^ These opportunities need to be leveraged towards improved health-care quality and UHC.

## Outcomes

The results of these systemic changes are reflected in improvements in health outcomes. The probability of dying between 15–50 years of age declined between 2000 and 2016 from 221 to 100 per 1000 women and 275 to 124 per 1000 men.^52^ Under-five and maternal mortality declined by 73% and 71%, respectively, between 1990 and 2019,^52^ and life expectancy at birth increased from 47 years to 65 years over the same period (Table 2).^66^ The incidence of tuberculosis declined by 61% between 2000 and 2019, while tuberculosis mortality declined 79% during the same period (Table 3). The number of acquired immune deficiency syndrome-related deaths dropped by 81% between 2000 and 2019.^53^^,^^58^^,^^67^

**Table 2:** Trends in health status, Ethiopia, 1990–2019

Health outcomes	Year					% change (1990–2019)
	1990	2000	2010	2019		
No. of live births	2 279 421	2 907 234	3 181 296	3 597 704		+58
Infant mortality, per 1 000 live births	123	97	59	43		−65
Under-five mortality, per 1 000 live births	205	166	88	55		−73
Maternal mortality, per 100 000 live births	1 400	871	676	412		−71
Life expectancy at birth, years	47	52	62	65		+38

Sources: Central Statistical Agency and ICF International; 2016.^52^ World Bank; 2019.^66^ Ethiopian Public Health Institute and ICF International; 2019.^53^

**Table 3:** Trend in key diseases incidence and mortality in Ethiopia, 2000–2019

Disease incidence and case fatality	Year				% change (2000–2019)
	2000	2010	2019		
Population in millions	66.2	87.6	112.1		+69
Tuberculosis incidence, per 100 000 population	421	268	164		−61
Tuberculosis-related mortality, per 100 000 population	112	39	24		−79
Malaria incidence, per 100 000 population	662	106	59		−91
Malaria-related mortality, per 100 000 population	28.4	3.2	3.2		−89
HIV incidence, no. of new infections per year	52 000	29 000	23 000		−56
AIDS-related deaths, no. per year	58 000	20 000	11 000		−81

AIDS: acquired immune deficiency syndrome; HIV: human immunodeficiency virus.

Sources: Assefa et al.; 2019.^58^ World Bank; 2019.^66^ Ethiopian Public Health Institute and ICF International; 2019.^53^ Joint United Nations Programme on HIV/AIDS; 2019.^67^

The average progress hides important inequities in health status among socioeconomic levels and regions and between urban and rural areas (Table 4). In 2016, under-five mortality was highest in Afar (125 per 1000 live births) and lowest in Addis Ababa (39 per 1000 live births).^52^ The under-five mortality rate ratio between regions and Addis Ababa increased between 2000 and 2016 in all regions except Tigray. Infant mortality was also higher among the children whose mothers had no education than those whose mothers had more than secondary education (64 and 35 per 1000 live births, respectively).^52^ Inequities in health outcomes can be explained by variations in the implementation of policies and strategies (including the health extension programme and community empowerment initiatives, health-systems strengthening, multisectoral actions) and health services delivery.^68^

**Table 4:** Under-five mortality per 1000 live births in regions in Ethiopia, 2000–2016

Region	Under-five mortality, per 1000 live births by year					Rate ratio in 2000	Rate ratio in 2016
	2000	2005	2011	2016			
Addis Ababa (Ref.)	114	72	53	39		1.0	1.0
Tigray	169	106	85	59		1.5	1.5
Dire Dawa	176	136	97	93		1.5	2.4
Amhara	183	154	108	85		1.6	2.2
Somali	184	93	122	94		1.6	2.4
Harari	191	103	94	72		1.7	1.8
Southern Nations, Nationalities and People’s region	192	142	116	88		1.7	2.3
Oromia	194	122	112	79		1.7	2.0
Benishangul-Gumuz	198	157	169	88		1.7	2.3
Afar	229	123	127	125		2.0	3.2
Gambela	233	156	123	88		2.0	2.3

Ref.: reference region.

Source: Central Statistical Agency and ICF International; 2016.^52^

## Discussion

Ethiopia has been consistently implementing the primary health-care approach to increase access to health services and improve the population’s health status over the past three decades. This has been possible due to country priorities and leadership, community engagement, the diagonal approach to disease control programmes, and health-systems strengthening and multisectoral action. The country’s leadership has set out pro-poor policies and strategies to achieve universal access to primary health-care services. Governance of the primary health-care system has been strengthened to improve planning, implementation, monitoring, harmonization and alignment of the different parts through a one-plan, one-budget, one-report approach. Despite the progress, key challenges remain towards UHC: inadequate coverage, inequity, slow health-systems transition, inadequate quality of care, and high out-of-pocket expenditure.

Similar findings in the implementation of the primary health-care approach were identified in other countries. In South Africa, re-engineering of the approach required health-systems strengthening, strong leadership and community empowerment.^69^ In India, implementation of primary health care improved service delivery for family planning, safe deliveries, immunization and health promotion.^70^ In Latin America, a commitment to renew primary health care as the basis of the health system had challenges in areas such as equity of access, quality of care, expanding coverage and preparing health systems for the ageing population.^71^

The challenges to implementation of primary health care in Ethiopia suggest that more of the same strategies or approaches will not be sustainable.^72^ The country needs to identify the groups with high disease burden or poor health services coverage, and adapt strategies to target these groups. We can learn from the response to HIV, which has demanded a targeted response and a differentiated care model providing services according to need.^73^ Similarly, the path towards UHC demands an approach that targets high-risk and vulnerable populations and locations. A precision public health strategy provides granular data to understand public health risks and customize interventions to more specific and homogeneous sub-populations.^74^

The epidemiological overlap between noncommunicable diseases and infectious diseases in Ethiopia demands a health-systems transition that integrates health services.^60^ Similar epidemiological overlaps were observed in other countries.^75^ Understanding of these dynamics is important to improve service delivery and achieve better health outcomes. A range of skills will be required, including interpersonal, teamwork, partnerships and collaboration with community groups and across different sectors, as well as resources to increase access and ensure continuity and quality of care. Again, the experience in scaling-up HIV care can be used to facilitate the health-systems transition to provide accessible, efficient and quality chronic care for patients with noncommunicable diseases.^76^^,^^77^

Provision of quality care is vital to improve utilization of health services, maintain continuity of care and ensure health. The current inadequate level of quality of care is a call for strengthened implementation of quality improvement initiatives in line with government plans identifying quality as part of its transformation agenda. It is commendable that the country has created a directorate dedicated to quality, and developed a strategy to improve the quality of health services.^78^ The lessons so far indicate that these initiatives will bring results if they are implemented consistently at all levels of the health system across the country.^64^

High out-of-pocket expenditure is a big challenge towards UHC in Ethiopia.^79^ Financing the health system towards UHC should reduce out-of-pocket expenditure through a prepayment mechanism. Increased government expenditure will be needed, funded through general taxation and social and community health insurance.^80^ Emerging evidence shows that people are willing to pay for the social health insurance scheme although there are still doubts about the benefit packages and quality of health services.^81^ However, implementation of these initiatives has been lagging; hence, there is a need for an accelerated and strengthened implementation of prepaid health financing mechanisms towards UHC. A deeper political commitment will be required, along with sustained community engagement and institutional capacity building.^44^

Health-systems strengthening (including infrastructure and human resources) has been essential for improving the health services and health status in Ethiopia. Further progress towards UHC will depend on enhancing community engagement, strengthened multisectoral action to improve living and working conditions, socioeconomic development and equitable distribution of resources. Community engagement needs to be complemented by political commitment to invest more in comprehensive primary health-care systems, address the social determinants of health, narrow inequities and advance UHC.^12^

Our analysis has both strengths and limitations. The main strengths are that it is a comprehensive study of the implementation of the primary health care in the country. It addresses a key knowledge gap on how to achieve UHC, which is the core target of SDG 3. It uses both quantitative and qualitative data to describe and analyse the implementation of primary health care. The main limitation is that our analysis was based on secondary data, and it may have the common limitations of secondary data, including quality. Nevertheless, these limitations are unlikely to affect our conclusions.

In conclusion, the implementation of the primary health-care approach has enabled Ethiopia to improve health services delivery towards UHC. However, more should be done to realize UHC by 2030. It is imperative that the country improves its domestic financing for health and that the health system targets high-risk, vulnerable and disadvantageous locations and populations through a precision public health approach. As the challenges are also driven by socioeconomic and political determinants, it is important to address them through the whole SDG agenda.
